# Effect of Simultaneous Immediate Implant Placement and Guided Bone Reconstruction with Ultra-Fine Titanium Mesh Membranes on Radiographic and Clinical Parameters after 18 Months of Loading

**DOI:** 10.3390/ma12101710

**Published:** 2019-05-26

**Authors:** Marco Tallarico, Francesco Mattia Ceruso, Leonardo Muzzi, Silvio Mario Meloni, Yong-Jin Kim, Marco Gargari, Matteo Martinolli

**Affiliations:** 1 DDS, Implantology and Prosthetic Aspects, Master of Science in Dentistry Program, Aldent University, Tirana, Albania. Private practice, 00100 Rome, Italy; 2 DDS, Department of Dentistry, “Fra G.B. Orsenigo-Ospedale San Pietro F.B.F.”, 00100 Rome, Italy; Hello_982@yahoo.it; 3 DDS, Private Practice, 53100 Siena, Italy; leo@leomuzzi.com; 4 DDS, School of Dentistry, University of Sassari, and private practice, 07021 Arzachena, Italy; melonisilviomario@yahoo.it; 5 DDS, Ilsan Apsun Dental Clinic, Ilsan 10381, Korea; ddskyj@naver.com; 6 DDS, Department of Clinical Science and Translational Medicine, University of Rome “Tor Vergata”, 00100 Rome, Italy; marco.gargari@gmail.com; 7 DDS, Private practice, 45014 Porto Viro, Italy; matteo.martinolli@hotmail.it

**Keywords:** titanium mesh, guided bone reconstruction, bone substitute, computer guided surgery

## Abstract

Background: The aim of the present prospective case series study was to evaluate the implant and prosthetic survival rates, complications and marginal bone loss using ultra-fine titanium mesh membrane with simultaneous implant placement, to provide space maintenance mandatory for guided bone reconstruction of alveolar bone defects. Materials and Methods: patients were recruited and treated at a private clinic in Rome, Italy, between March 2016 and October 2017. Self-tapping tapered implants were placed through a computer-guided template-assisted approach. Autogenous bone was placed alone over the exposed implant surface, then mixed with inorganic bovine bone material. Finally, the membrane was connected and shaped in order to securely enclose the graft area, and the healing cap was connected and screwed onto the height connector. Outcome measures were: implant and prosthetic failure, biological and mechanical complications, marginal and volumetric bone level changes, esthetic evaluation performed according to the pink aesthetic score (PES). Results: in total, seven patients (five women, two men) with a mean age of 52.7 ± 20.3 years (range: 27–71) received 10 self-tapping tapered implants and simultaneous guided bone regeneration with ultra-fine titanium mesh membranes. No implants and no prostheses failed during the entire follow-up period. One slightly membrane exposure was observed one month after implant placement in one patient. The mean marginal bone loss (MBL) at implant loading was 0.13 ± 0.09 mm (95% CI 0.08–0.19). At the 18-month follow-up examination, the mean MBL was 0.28 ± 0.33 mm (95% CI 0.07–0.50) The difference was not statistically significant (0.15 ± 0.31; 95% CI 0.05–0.35; P = 0.1888). The mean horizontal alveolar ridge width was 3.72 ± 1.08 mm (95% CI 3.22–4.22 mm). At the II-stage surgery, the mean bone width was 8.79 ± 0.98 mm (95% CI 8.51–9.07 mm). The mean bone gain was 5.06 ± 1.13 mm (95% CI 4.68–5.44 mm; P = 0.000). The mean volume of the grafted bone calculated using the superimposition technique was 0.99 ± 0.38 CC (95% CI 0.75–1.23 CC). The mean PES at implant loading was 8.2 ± 0.8 mm (95% CI 7.7–8.7). At the 18-month follow-up examination, the mean PES was 12.0 ± 0.7 mm (95% CI 11.5–12.5) The difference was statistically significant (3.8 ± 0.4; 95% CI 3.5–4.1; P = 0.0000); Conclusion: with the limitation of the present prospective study, the guided bone reconstruction using an ultra-fine titanium mesh membrane with simultaneous implant placement seems to provide good and stable results in implant/prosthesis success. Further research with a longer follow-up and a higher sample size are needed to confirm the results from this preliminary report.

## 1. Introduction

Alveolar bone atrophy is a chronic and progressive clinical situation characterized by moderate to severe loss of bone volume due to teeth loss or extraction [1]. Moreover, local conditions or diseases, such as, traumatic extraction, periodontal disease, and trauma, could magnify this pathological condition, making dental implants placement difficult or unfavorable from both a functional and aesthetic perspective [2]. To overcome these possible drawbacks, bone reconstruction techniques have been introduced. Among these, guided bone regeneration (GBR) is a surgical procedure that uses a graft material as a scaffold [3,4] isolated and protected with a membrane, from the non-osteogenic cells, derived from the adjacent connective tissue. Thereby, the barrier effect of the membrane should permit only to the osteogenic cells, derived from the surrounding bone and vessels, to move into the bone defect allowing for bone formation through the presence of stimulating signals.

Both resorbable and non-resorbable membranes have been used to isolate and maintain a correct and planned biological scaffold, needed for the formation of new bone tissue. Given the nature of their function, the non-resorbable membrane should be more predictable and safer to use. However, the main limitation is that an adjunctive surgery is necessary to remove the membrane. Nevertheless, guided bone reconstruction is usually performed in two stages or with a submerged implant protocol when performed one-stage. So, the membrane could be removed at implant placement or at second stage if GBR was done at same time as the implant.

In daily practice, expanded-polytetra-fluoroethylene (e-PTFE) non-resorbable membranes could be considered as the first choice in the reconstructions of horizontal and vertical bone defects [5,6,7,8] Nevertheless, since the 1990s, various researchers investigated the clinical use of ultra-fine titanium mesh (Ti-meshes) for reconstruction of the atrophic mandible and maxilla [9] The titanium is more resistant to collapse than e-PTFE and resorbable membranes [5,6]. Hence, the rigidity of the titanium may work like a scaffold, maintaining the space required for the bone regeneration, even in cases of a large bone defect, such as vertical bone reconstruction.

The porosity of the Ti-meshes can be modified to achieve better tissue integration and formation. Varying the porosity of the Ti-meshes allows fluid and nutrients to pass through the membrane while avoiding the infiltrating cells.

Nowadays, different shapes of Ti-mesh membrane have been proposed to maximize new bone formation, stabilize the graft materials under the membrane, and reduce the risk of membrane exposure, that can provide collapse and/or ingrowth of the adjacent soft tissue [10,11].

The aim of the present prospective case series study was to evaluate the implant and prosthetic survival rates, complications, marginal bone loss, using ultra-fine titanium mesh membrane and simultaneous implant placement, to provide space maintenance necessary for bone augmentation of alveolar bone defects. This trial followed the provisions of the Strengthening the Reporting of Observational Studies in Epidemiology (STROBE) statement.

## 2. Materials and Methods

This research was designed as a case series study to evaluate the clinical and radiographic outcomes of simultaneous guided bone reconstruction and implant placement with ultra-fine titanium mesh membrane. Patients were recruited and treated at a private clinic in Rome, Italy, between March 2016 and October 2017. The surgical procedures were performed by an expert clinician (MT) certified in implant-based therapy at the European association for osseointegration in 2013. All patients were informed about the nature of the study and gave their written consent for surgical and prosthetic procedures and for the use of radiologic and clinical data for publication. The principles embodied in the Helsinki Declaration of 2013 were adhered strictly. Moreover, the radiological protocol was approved by the Scientific Technical and Ethical Committee of the University of Sassari (2069/CE).

Any consecutive patients aged 18 years or older who presented with partial edentulism of the maxilla or mandible, able to understand and sign an informed consent, and requiring an implant supported restoration, were considered eligible for inclusion in this study. Patients were finally included if presented with a Cawood and Howell Class IV to VI atrophy of the residual alveolar ridge at the cone beam computed tomography examination. Patients were excluded if they presented with general contraindications to implant surgery (such as irradiation of the head and neck area within the previous five years before implantation, or uncontrolled diabetes); pregnancy or lactation; substance abuse; psychiatric therapy or unrealistic expectations; previous or ongoing treatment with intravenous bisphosphonates; untreated periodontitis od poor oral hygiene (bleeding on probing and/or plaque index ≥ 25%); heavy smokers (≥11 cigarettes/day); post-extractive sites.

Before implant surgery, a CBCT scan (CBCT, CRANEX 3D; Soredex, Tuusula, Finland) and an intraoral digital impression (CS 3600 intraoral scanner, Carestream Dental, Atlanta, GA, USA) were taken. The CBCT scan was set with a field of view of 80 mm × 150 mm; voxel size of 0.3 µm; 90 kV; 6.3–10 mA for 4.5 s, resulting in a dose-area product of 579.7–920.2 (mGycm^2^). The STL and DICOM (Digital Imaging and COmmunications in Medicine) data were imported in a dedicate software for diagnosis and implant planning (3Diagnosys version 4.2, 3DIEMME srl, Cantù, Italy). Afterwards, prosthetic-driven implants were virtually planned.

One hour prior to surgery, a single dose of antibiotics (2 g of amoxicillin and clavulanic acid, or 600 mg of clindamycin if allergic to penicillin) was administered prophylactically. A 0.2% chlorhexidine digluconate mouth rinse was administered for two minutes prior to surgery.

All the patients were treated under oral sedation with diazepam 10 mg (Valium, Roche S.p.A., Monza, Italy). Local anesthesia using articaine with adrenaline 1:100,000 was administered. A midcrestal incision was made into the keratinized tissue using surgical scalpel blade No. 15C. A full-thickness flap was elevated beyond the mucogingival junction. Then, two vertical incisions were made one tooth away from the bone defect, or at least 5 mm away in case of edentulous area. Then, the recipient site was cleaned by removing all the soft tissue remnants. Self-tapping tapered TSIII implant (Osstem Implant, Seoul, Korea) was placed through a computer-guided template-assisted approach, at the bone level or 1 mm deeper, according to the drilling protocol suggested by the manufacturer. All the surgical templates were teeth-supported and were made without metallic sleeves [12,13]. The main characteristic of the OssBuilder membrane is that it is fixed directly to the implant in a one-stage approach. To ensure the membrane onto the implant, a special tool named height connector (Osstem Implant) must to be used. This tool is available in different height to connect the membrane at the implant at different level depending on the residual bone and implant depth. Using a 1.2 hex driver, the height connector was screwed to the fixture at a 5 to 8 N·cm. Then, autogenous bone was harvested from the adjacent area, using a minimally invasive cortical bone collector (Micross, Meta, Italy). The bone was harvested in the area mesial or distal close to the defect. Afterwards, the bone defect was measured to determinate the appropriate shape and size of the titanium mesh membrane (OssBuilder, Osstem Implant). All the used membranes were of pure titanium grade 2 and designed with 0.6 to 1 mm diameter pores.

**Figure 1:**
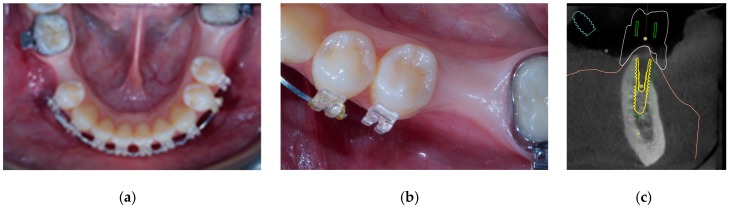
Pre-clinical scenario and virtual planning. Occlusal view of the edentulous area (**a**,**b**). Virtual implant planning (**c**).

**Figure 2:**
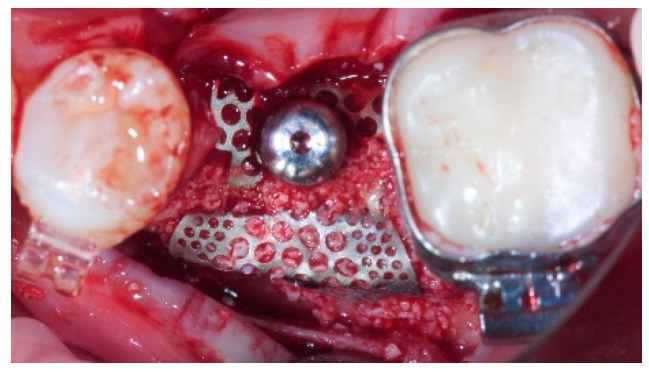
Clinical view at implant placement. The ultra-fine titanium mesh after bone graft. The healing cap was used to anchor the titanium mesh to the height connector.

**Figure 3:**
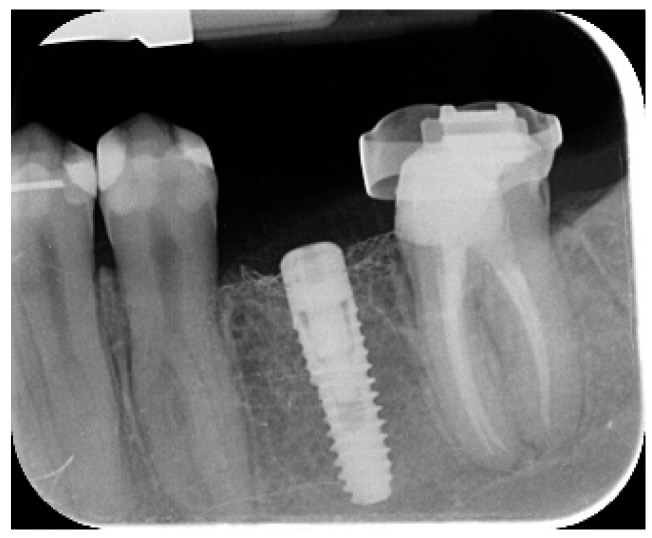
Radiographic view at implant placement.

Autogenous bone was placed alone over the exposed implant surface. Then, a second layer composed by autogenous bone mixed with anorganic bovine bone material (Bio-OSS, Geistlich Biomaterials Italia S.r.l.) in a 1:1 ratio, was used to graft the remained defect. The bone graft was over contoured to compensate final graft resorption. Finally, the membrane was connected to the height connector and shaped in order to securely enclose the graft area, and the healing cap was connected and screwed onto the height connector using the cover cap driver at 5 to 8 N·cm. A periosteal incision was made to allow a passive, tension-free adaptation and clousure of the flap. The wound was sutured in two layers with a 4-0 polyglactin 910 suture (Vicryl V271; Ethicon, West Somerville, NJ, USA) (Figure 1, Figure 2 and Figure 3). Antibiotic coverage was given for seven days (1 g of amoxicillin and clavulanic acid or 300 mg of clindamycin twice a day) after surgery. A 0.2% chlorhexidine digluconate mouth rinse was prescribed for one minute, twice a day, for three weeks, and a soft diet was recommended for four weeks. Ibuprofen 400 mg (or paracetamol 1 g) was to be taken in the event of pain.

**Figure 4:**
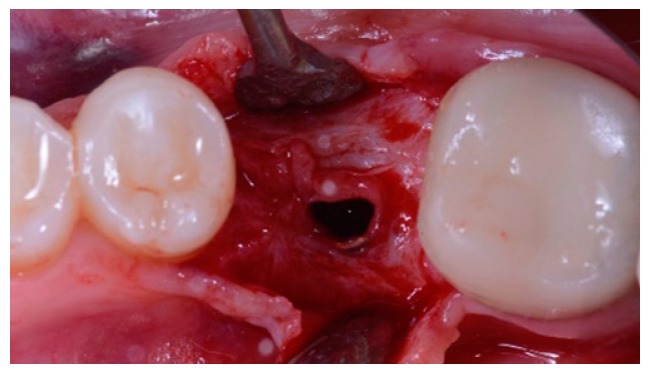
Implant reopening. Bone gain obtained after guided bone reconstruction.

**Figure 5:**
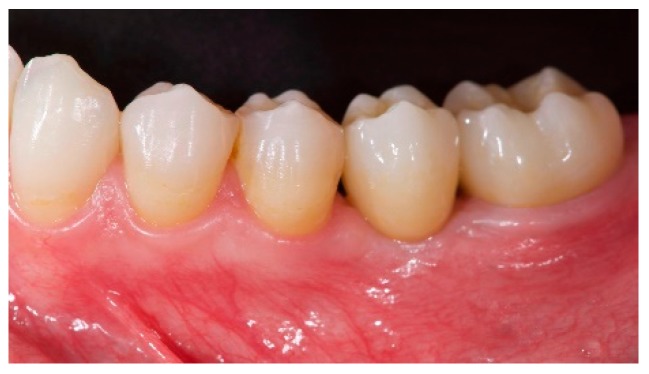
Definitive prosthesis delivery.

**Figure 6:**
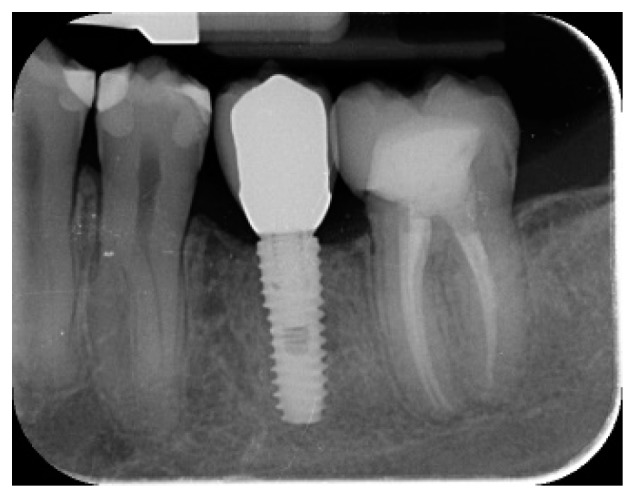
Radiographic view after prosthesis delivery.

**Figure 7:**
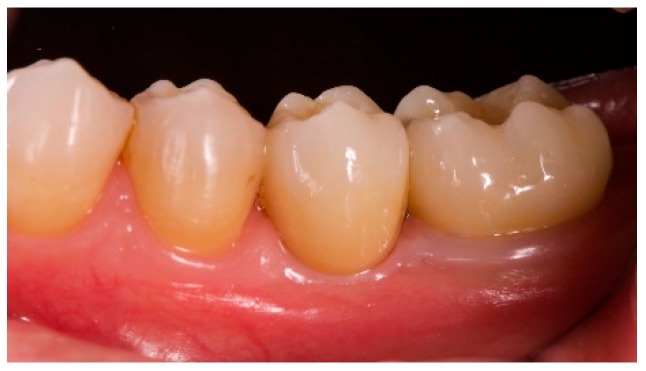
18 months clinical follow-up.

**Figure 8:**
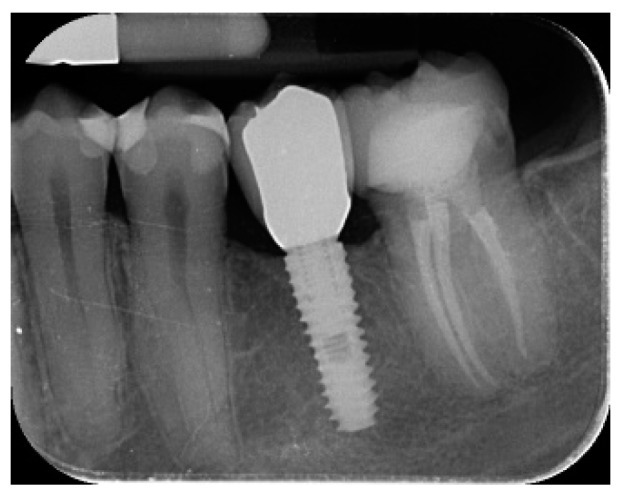
18 months radiographic follow-up.

Seven to eight months after implant placement, the patient underwent a second CBCT scan to evaluate the bone reconstruction. The CBCT scan was set with a field of view of 60 mm × 80 mm; voxel size of 0.3 µm; 90 kV; 4–6.3 mA for 2.3 s, resulting in a dose-area product of 192.4–307.8 (mGycm^2^). Then, a second-stage surgical procedure was performed, making sure to preserve the keratinized tissue around the dental implant. The ultra-fine titanium mesh was carefully removed and a healing abutment was screwed onto the fixture (Figure 4). A platelet-rich fibrin (PRF) membranes was then adapted over the reconstructed bone and the flap was drawn coronally and sutured. Two weeks later, a provisional restoration was provided. Three to four months later, a CAD/CAM screw-retained zirconia restoration was delivered (Figure 5 and Figure 6). The occlusion was adjusted to avoid premature contacts. Periapical radiographs and clinical photographs were taken. Follow-up visits were scheduled every three months after implant placement (Figure 7 and Figure 8).

## 3. Outcomes Measures Included:

**Figure 9:**
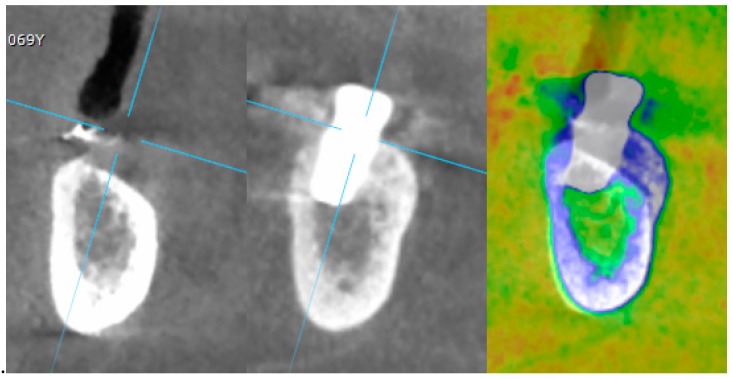
Superimposition of pre- and seven to eight months post-operative CBCT scan.

- Implant survival rate: an implant was considered a failure if it presented any mobility, implant fracture or an infection that mandated implant removal.
- A restoration was considered failed if it needed to be replaced by an alternative restoration.
- Presence of biological (pain, swelling, suppuration, etc.) or mechanical (screw loosening or fracture of the framework and/or the veneering material, etc.) complications.
- Marginal bone level changes were assessed by digital periapical radiographs (Digora Optime; Soredex, Tuusula, Finland) using the parallel technique and commercially available film holders. Three time points were evaluated, at implant placement (baseline), immediately after the insertion of the restoration, and one year after loading. The averaged mesial and distal distances from the most coronal margin of the implant and the first bone-to-implant contact was measured to the nearest 0.01 mm and taken as the marginal bone level. The difference in levels between time points was taken as marginal bone loss (MBL).
- Horizontal bone augmentation was evaluated at the CBCT scans, 1 mm below the original bone crest. The volumetric data were superimposed using the adjacent teeth as reference points, and a new generated set of DICOM data was stored as a separately files. Measures were taken before and after the treatment, and the difference of these two measurements was taken as horizontal bone augmentation (Figure 9).
- Volumetric measurements of the reconstructed bone was performed automatically on the merged CBCT set of volumetric data, using the Fusion module of the OnDemand 3D software (Cybermed Inc., Seoul, Korea), according a previously published protocol [8].
- The aesthetic evaluation was performed according to the pink aesthetic score (PES) on the vestibular and occlusal pictures taken including at least one adjacent tooth per side. The values were assessed at 6 and 12-months after loading follow-up examinations (18-months follow-up) [14] Seven variables (mesial papilla, distal papilla, soft-tissue level, soft-tissue contour, alveolar process deficiency, soft-tissue color and texture) were assessed with a 2-1-0 score (2 being best and 0 being poorest) by the same blinded dentist.

Implant and prosthetic surgical rates and complications were assessed by the same clinicians that performed all the cases (MT). Marginal bone loss and pink esthetic score were evaluated by any independent assessor not previously involved in the study (MM). Descriptive analysis was performed for mean ± standard deviation (SD), median, and 95% confidence interval (CI) using Numbers (Version 5.2) for Mac OS High Sierra 10.X. Comparisons between follow-ups were made by a paired student t-test using SPSS (Version 22.0; IBM Corporation, Armonk, NY, USA) for Mac OS High Sierra 10.X. All statistical comparisons were conducted at a 0.05 level of significance. The statistical unit was the implant.

## 4. Results

In total, seven patients (five women, two men) with a mean age of 52.7 ± 20.3 years (range: 27–71) received 10 self-tapping tapered TSIII implants (Osstem Implant) and simultaneous guided bone regeneration with ultra-fine titanium mesh membranes (OssBuilder; Osstem Implant). 18 months after loading, no patients dropped-out and no deviation from the original protocol occurred. The mean follow-up time was 20.85 ± 42.84 months after implant placement (range: 18–24 months). All implants were inserted at torques between 35 and 45 N·cm using a computer-based, template-guided approach (Table 1).

No implants and no prostheses failed during the follow-up period. One slight membrane exposure was observed one month after implant placement in one patient. Patients were instructed to carefully brush the membrane with a soft toothbrush and to apply chlorhexidine spray twice a day. Complete soft tissue healing was observed four weeks later.

All the implants were place at bone level or 1 mm deeper. The mean marginal bone loss at implant loading was 0.13 ± 0.09 mm (95% CI 0.08–0.19). At the 18-month follow-up examination, the mean marginal bone loss was 0.28 ± 0.33 mm (95% CI 0.07–0.50). The difference was not statistically significant (0.15 ± 0.31; 95% CI 0.05–0.35; P = 0.1888) (Table 2).

The mean horizontal alveolar ridge width was 3.72 ± 1.08 mm (95% CI 3.22–4.22 mm). At the II-stage surgery, the mean bone width was 8.79 ± 0.98 mm (95% CI 8.51–9.07 mm). The mean bone gain was 5.06 ± 1.13 mm (95% CI 4.68–5.44 mm; P = 0.000). The mean volume of the grafted bone calculated using the superimposition technique was 0.99 ± 0.38 CC (95% CI 0.75–1.23 CC).

The mean PES at implant loading was 8.2 ± 0.8 mm (95% CI 7.7–8.7). At the 18-month follow-up examination, the mean PES was 12.0 ± 0.7 mm (95% CI 11.5–12.5) The difference was statistically significant (3.8 ± 0.4; 95% CI 3.5–4.1; P = 0.0000) (Table 2).

## 5. Discussion

The present research was conducted to evaluate clinical and radiologic data, one year after loading, of a guided bone regeneration using ultra-fine titanium mesh membrane with simultaneous implant placement, to provide a scaffold effect (space maintenance) that is necessary to allow for bone reconstruction of crestal bone defects. Because this research had been designed as a prospective case series study, its primary limitation is the lack of a control group. The main limitation of the present study was the small sample size. Nevertheless, this research was designed as a prospective case series study. This limitation could be solved by performing further trials with larger sample sizes, that could be calculated based on the preliminary results of the present study. Another limitation of this study, which could be a confounding factor, was that the gingival biotype was not considered. Nevertheless, the results of the present study were in agreement with previously published reports [7,8]. In fact, several researchers supported the ideal mechanical properties of the Ti-mesh in terms of rigidity, plasticity, and elasticity [15]. Furthermore, Ti-mesh seems to be less susceptible to bacterial contamination, compared to resorbable materials [16]. To underline this concept, authors observed only one slight membrane exposure one month after implant placement. Patients were instructed to carefully brush the membrane with a soft toothbrush and to apply chlorhexidine spray twice a day. Complete soft tissue healing was observed four weeks later. This minor complication suggests the effectiveness of this titanium mesh. Furthermore, in this prospective study no implants and no prostheses failed during the follow-up period. Thanks to this data it is reasonable to consider this procedure safe and easy for the clinician. Several characteristics of barrier membranes are necessary for successful guided bone reconstruction, such as clinical manageability, biocompatibility, capability for space creation, cell exclusion, and tissue integration [17]. Ti-meshes are non-resorbable membranes, and their porosity can be varied to achieve tissue compatibility [3]. In the present study, pure titanium grade 2 ultra-fine membrane was used. The thickness of the titanium mesh was 100 µm, while the porosity was 0.6 to 1 mm diameter. The pores of 1 mm diameter may provide optimal blood supply and growth factors diffusion, necessary for the promotion of healing and bone regeneration. However, pores of 0.6 mm diameter may prevent shifting or migration of bone grafting material while allowing for blood supply diffusion. Furthermore, these membrane presented some side perforations that should be able to maintain high mechanical strength, while also allowing flexibility for shaping and modification.

Nonetheless, membrane exposure and infection are relatively common [18,19,20] In contrast to PTFE membrane which is relatively thick, approximately 200 μm, and susceptible to bacterial entrapment due to its porous structure, titanium can be manufactured in a thinner form [21].

Recently, ultra-fine surface patterns associated with pores, channels, and other features in bioscaffolds are known to influence cell migration, proliferation, and differentiation. Thus, laser processing technology is favorable to create a variety of ultra-fine surface patterns [22].

To obtain a good and predictable bone regeneration, the bone graft should include three characteristics:

(1) Osteoblasts cells or mesenchymal stem cells; (2) Growth factors, to ensure regeneration progress; and (3) A ‘skeleton’ able to offer mechanical support for cell adherence and proliferation [23].

The authors hypothesized that this ultra-fine structure, used in this prospective study, allows for the minimization of soft tissue ingrowth into the micro perforation and keeps the permeability of nutrients or tissue fluid across the membrane, which would promote attachment, migration, and proliferation of bone marrow–derived cells, resulting in more bone regeneration.

During the last decade, long-term studies have evaluated and underlined that GBR is a successful and predictable technique for the clinician to obtain vertical and horizontal ridge augmentation [1]. GBR can be achieved with two different approaches: application of either a polytetrafluoroethylene (PTFE) titanium-reinforced membrane (i.e., a non-resorbable membrane) and an ultra-fine titanium mesh or a collagen membrane (i.e., a resorbable membrane) [1,8,22,24,25]. Implants were placed using a computer-guided template-assisted approach, at the bone level or 1 mm deeper, according to the drilling protocol suggested by the manufacturer [12,13]. In our opinion this guided approach allows for a better prosthetically driven implant installation and permits bone regeneration in accordance with the prothesis needed.

The results of the present study are in agreement with recent literature published on implant placed after or during GBR procedure [1,2,3,4,5,6,7].

## 6. Conclusions

With the limitation of the present prospective study, the guided bone reconstruction using an ultra-fine titanium mesh membrane with simultaneous implant placement, to provide space maintenance necessary for bone augmentation of alveolar bone defects seems to provide implant/prosthesis success. Further research with a higher sample size and longer follow-up are needed to confirm these preliminary results.

## Figures and Tables

**Table 1:** Main patient and implant characteristics.

**Age (years)**	52.7 ± 20.3
**Mean Follow-up (months)**	20.85 ± 42.84
**Female patients**	5
**Male patients**	2
**Implant placed**	10
**Complications**	1
**Implant failure**	0
**Prosthesis failure**	0
**Insertion torque**	40 ± 5

**Table 2:** Implant follow-up.

	Implant Loading	18-Month Follow-Up
Mean marginal bone loss (mm)	0.13 ± 0.09 (95% CI 0.08–0.19)	0.28 ± 0.33 (95% CI 0.07–0.50)
Mean horizontal alveolar ridge (mm)	2.92 ± 0.48 (95% CI 2.68–3.16)	8.29 ± 2.14 (95% CI 7.59–8.99)
PES (mm)	8.2 ± 0.8 (95% CI 7.7–8.7)	12.0 ± 0.7 (95% CI 11.5–12.5)
